# Theoretical Investigation of Iridium Complex with Aggregation-Induced Emission Properties

**DOI:** 10.3390/molecules29030580

**Published:** 2024-01-24

**Authors:** Piotr Lodowski, Maria Jaworska

**Affiliations:** Institute of Chemistry, University of Silesia in Katowice, Szkolna 9, 40-006 Katowice, Poland; piotr.lodowski@us.edu.pl

**Keywords:** AIE, iridium complex, DFT, TD-DFT, RASTC

## Abstract

The mechanism of aggregation-induced emission (AIE) for the bis(1-(2,4-difluorophenyl)-1H-pyrazole)(2-(20-hydroxyphenyl)-2-oxazoline)iridium(III) complex, denoted as Ir(dfppz)_2_(oz), was investigated with use DFT and the TD-DFT level of theory. The mechanism of radiationless deactivation of the triplet state was elucidated. Such a mechanism requires an additional, photophysical triplet channel of the internal conversion (IC) type, which is activated as a result of intramolecular motion deforming the structure of the oz ligand and distorting the iridium coordination sphere. Formally, the rotational movement of the oxazoline relative to the C–C bond in the oz ligand is the main active coordinate that leads to the opening of the triplet channel. The rotation of the oxazoline group and the elongation of the Ir-N_ox_ bond cause a transition between the luminescent, low-lying triplet state with a d/π→π * characteristic ( $T_{1(\mathrm{eq})}$ ), and the radiationless d→d triplet state ( $T_{1(\mathrm{Ir})}$ ). This transition is made possible by the low energy barrier, which, based on calculations, was estimated at approximately 8.5 kcal/mol. Dimerization, or generally aggregation of the complex molecules, blocks the intramolecular movement in the ligand and is responsible for a strong increase in the energy barrier for the $T_{1(\mathrm{eq})}⇝T_{1(\mathrm{Ir})}$ conversion of triplet states. Thus, the aggregation phenomenon blocks the nonradiative deactivation channel of the excited states and, consequently, contributes to directing the photophysical process toward phosphorescence. The mechanism involved in locking the nonradiative triplet path can be called restricted access to singlet–triplet crossing (RASTC).

## 1. Introduction

Aggregation-induced emission (AIE) is a phenomenon that involves the increased luminescence of a chemical compound in the aggregates or in the solid state compared to in a solution where it shows no or weak emission [1,2,3,4,5,6,7,8,9]. Tang et al. first described this phenomenon for siloles [10,11]. Since then, many compounds have been found with such properties. Other terms for this phenomenon have also been used, such as aggregation-induced enhanced emission or aggregation-induced enhanced phosphorescence (AIEE and AIEP, respectively). The molecules with AIE properties are generally named AIE luminogens (AIEgens). In compounds with AIE properties, other phenomena, such as mechanochromism or vapochromism [2,12,13,14,15], often occur. These allow for very wide practical applications of this type of system. Examples of possible applications of AIEgen materials include anticounterfeiting fluorescent hydrogels and inks, the luminescent detection of explosives, medical bioimaging, data recording, encryption, and OLED materials [16,17,18,19,20,21,22].

Several possible mechanisms have been proposed that lead to the occurrence of AIE. Blocking channels for nonradiative deactivation of excited states in aggregates or the solid state may be one of the causes of AIE. Restricting the possibility of rotation or other intramolecular motions, for which the terms restriction of intramolecular rotations (RIR) or restriction of intramolecular motions (RIM) [23,24,25,26] are used, is one of the possible mechanisms of AIE. Based on experimental findings and theoretical calculations, other mechanisms of the AIE phenomenon, such as restriction of access to conical intersection (RACI), restriction of vibronic coupling (RVC), restriction of access to the dark state (RADS), and suppression of photochemical reaction (SPRC) [15,23,24,25,27,28,29,30,31,32,33,34], have also been proposed. All of them can be traced to the geometric changes caused by the rigidification of the molecular system in the aggregated or solid state; hence, they can be regarded as the consequences of RIM.

In addition to organic AIEgens, metal complexes are also subject to intense interest in terms of AIE properties [13,14,35,36,37,38]. Organic AIEgens are fluorescent materials, while their organometallic counterparts exhibit phosphorescence during light emission. Compounds characterized by phosphorescence have unique properties due to the involvement of triplet excited states. Phosphorescent materials (PMCs) have great advantages, such as long emission times and large Stokes shifts, and could be used in a wide range of practical applications; therefore, there is great interest in organometallic AIEgens [39,40,41,42,43].

**Figure 1:**
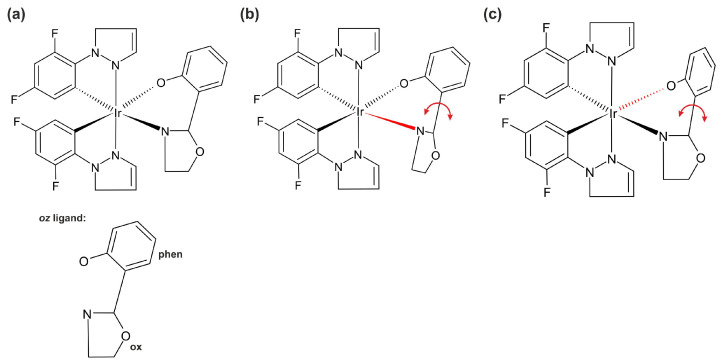
(**a**) Molecular structure of bis(1-(2,4-difluorophenyl)-1H-pyrazole)(2-(20-hydroxyphenyl)-2-oxazoline)iridium(III) complex, denoted as Ir(dfppz)_2_(oz) (**1**), where *oz* is 2-(20-hydroxyphenyl)-2-oxazoline group. (**b**) Rotation of the oxazoline group. (**c**) Rotation of the phenolate group.

In this work, we investigated the iridium(III) complex, bis(1-(2,4-difluorophenyl)-1H-pyrazole)(2-(20-hydroxyphenyl)-2-oxazoline)iridium(III) (denoted further as **1**), which we refer to hereafter as Ir(dfppz)_2_(oz), (Figure 1). Complex **1** shows AIE in acetonitrile solutions, in which it is soluble. The phosphorescence intensity is low, but, after adding water, the intensity increases due to the formation of aggregates [44,45]. According to theoretical and experimental studies, the increased luminescence of organometallic AIEgens in aggregates and solids is associated with substituents in the ligands, whose rotation is inhibited under these conditions [43,46,47,48]. In complex **1**, the ligands do not have any rotatable side groups, Therefore the question about the underlying mechanism is particularly interesting. To explain the source of AIE in this compound, we performed theoretical calculations for the monomer and for the dimer of **1**, the latter being a model of the state in which the complex forms aggregates. Our goal was to determine what types of molecular motions contribute to the AIE effect.

## 2. Results

### 2.1. Geometry

**Figure 2:**
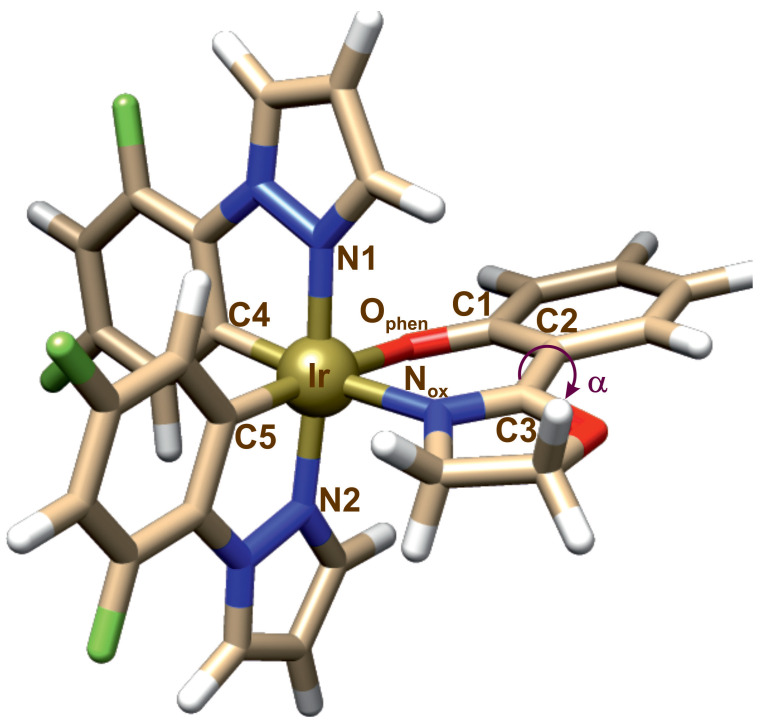
Optimized structure of complex **1**.

The optimized geometry of complex **1** is presented in Figure 2, and the calculated structural parameters are gathered in Table 1. As can be seen in Table 1, there is a very good agreement between the calculated and experimental geometry parameters.

### 2.2. Electronic Spectrum of **1**

**Figure 3:**
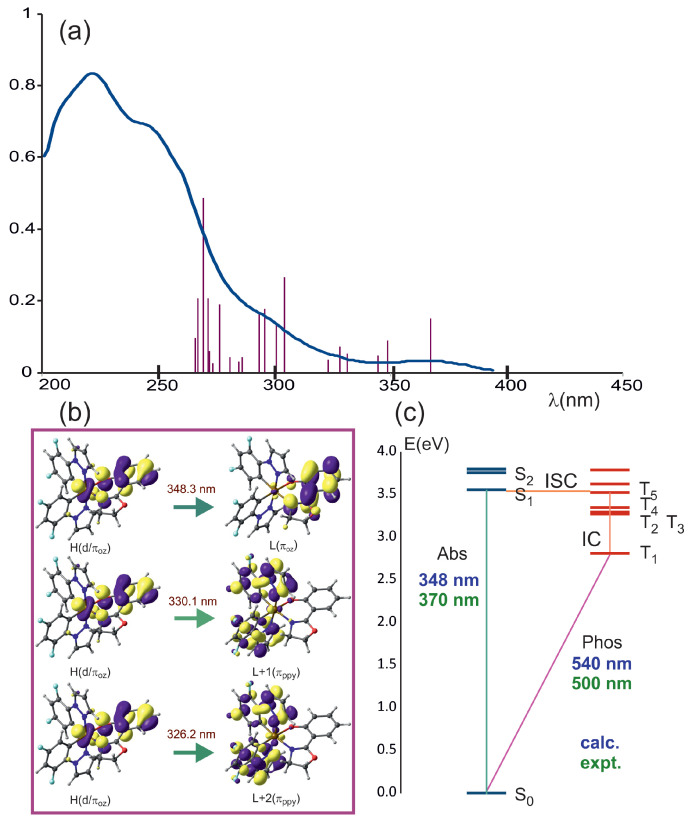
(**a**) Blue solid line—experimental absorption spectrum from [44], bars—calculated electronic transitions. Calculated transitions are shifted by 20 nm toward longer wavelengths. (**b**) Orbital excitations for the three lowest energy electronic transitions. (**c**) Energy of the lowest singlet and triplet states of the complex **1**. Experimental (green) and calculated (blue) wavelengths of the absorption and phosphorescence emission are shown. ISC and IC stand for intersystem crossing and internal conversion, respectively.

The TD-DFT method was used to describe the electronic excited states of the studied complex. Figure 3a presents the obtained results in the form of the spectral distribution of electronic excitations. Additionally, for comparison, a reproduction of the spectral line of the experimental absorption spectrum is included. Because the calculated transitions are at shorter wavelengths than the experimental ones, the calculated wavelengths were shifted 20 nm toward the longer wavelengths to fit them to the experimental data and to better illustrate the relationship between the calculated and experimental spectra. It should be noted that the differences between the experimental bands and those assigned to them from the theoretical calculations are within 20 nm or less, which is very good agreement. In Figure 3b,c, the MOs of the three lowest energy electronic transitions and an energy diagram of the lowest singlet and triplet states are presented, respectively.

Several lowest excited singlet states are collected in Table 2. All calculated singlet and triplet states are provided in Tables S1 and S2. The three lowest energy calculated transitions presented as vertical bars in Figure 3a can be assigned to the experimental band with a maximum at about 370 nm [44]. The lowest energy one at 348 nm has a $d/\pi_{oz}\to \pi_{oz}^{*}$ character. The nature of the lowest triplet state is the same as that of the lowest singlet (Table S2). This triplet determines the photophysical properties of complex **1**. The next two transitions are of the $d/\pi_{oz}\to \pi_{dfppz}^{*}$ type. The subsequent several calculated transitions between 313 and 283 nm, which can be attributed to the experimental bands at 325 and 290 nm, are of similar nature. At higher energies, the transitions of the $\pi_{dfppz}\to \pi_{dfppz}^{*}$ type appear, which are responsible for bands with higher intensity. The calculated transition at 252 nm can be assigned to the experimental band at 260 nm (Table S1). This is a typical situation, where π→π * transitions are very intense.

In Table 2 and Figure 3c, the calculated wavelength of phosphorescence is shown. It amounts to 540 nm, compared to the experimental 500 nm [44]. The phosphorescence comes from the LUMO to HOMO transition with the same character as the lowest electronic transition in the absorption spectrum.

### 2.3. Properties of Monomer Complex ***1***

In the monomeric form of complex **1**, there are no rotatable substituents in the ligands. The phosphorescence quenching mechanism for the monomer should therefore be different than is usually proposed for this mechanism. We took into account a possibility in which changes in geometry directly in the coordination sphere of the metal affect the photophysics of complex **1**, which determines its AIE properties. We decided to check the effect of geometry changes inside the *oz* ligand, namely, rotation around the bond between phenolate and the oxazoline motifs. Formally, such a rotation corresponds to a change in the selected dihedral angle in the structure of the ligand (Figure 2, angle α (C1-C2-C3-N_*ox*_)). Since the *oz* ligand is bound to the iridium ion, there are two possibilities: rotation of the oxazoline or the phenolate moiety. In these cases, the ligand–metal bond is broken with the nitrogen or oxygen atom, respectively.

Compounds of iridium and other metals of the second and third transition row exhibit phosphorescence from the triplet state due to the rapid singlet–triplet transition. Taking this into account, we determined the potential energy curve of the lowest triplet state as a function of the dihedral angle for the rotation of the oxazoline and phenolate units.

#### 2.3.1. Rotation of the Oxazoline Moiety

**Figure 4:**
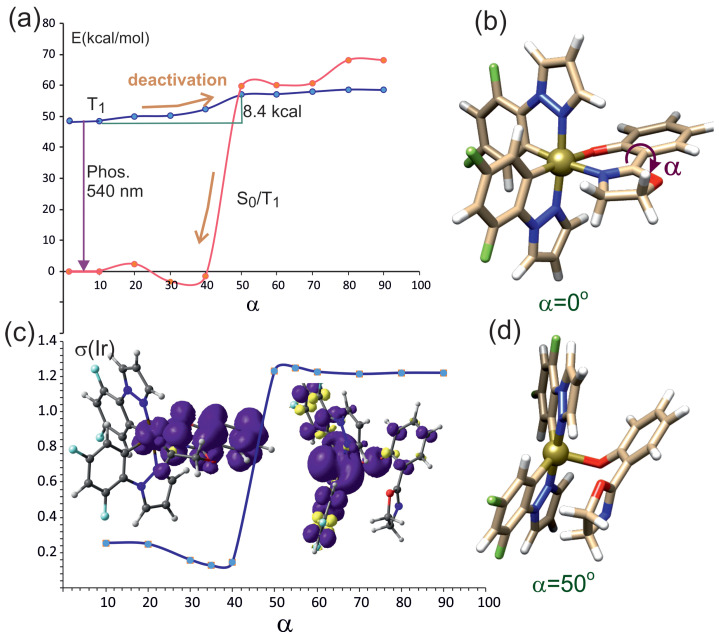
(**a**) Energy as a function of rotation angle around the oxazoline–phenolate bond, where the oxazoline moiety is being rotated. T_1_ (blue line)—energy of optimized-geometry lowest triplet state, S_0_/T_1_(red line)—energy of the lowest singlet state at the geometry of triplet T_1_. (**b**) Complex **1**, with rotation angle α marked. (**c**) Spin population value in the T_1_ state of **1** on the Ir ion as a function of angle α . The spin density contours of complex **1** are drawn at α equal 10° and 50°. (**d**) Geometry of **1** at rotation angle of 50° showing broken Ir-N_*ox*_ bond.

Figure 4a presents the potential energy curves as a function of the rotation angle α for the lowest triplet state, T_1_ (blue line), and the lowest singlet state S_0_ calculated at the T_1_ geometry (red line). In the case considered here, the rotation relative to the the oxazoline-phenolate bond (Figure 4b, α rotation angle) corresponds to a change in the position of the oxazoline motif, and, at the same time, it is associated with a change in the Ir-N_*ox*_ distance. As the value of the rotation angle increases, this distance is increased until the Ir-N_*ox*_ bond is broken. The dependence of the Ir-N_*ox*_ bond distance on the rotation angle is depicted in Figure S2.

According to the shape of the PEC presented in Figure 4a, due to rotation, the energy of the T_1_ state increases up to angle of approximately 50° and then remains practically unchanged. The same changes in the value of the rotation angle cause a rapid change in the energy of the singlet state, S_0_. The energy of S_0_ calculated at T_1_ geometry increases rapidly at about 40° and intersects with T_1_. The crossing point on T_1_ curve is placed at 8.4 kcal above the T_1_ minimum. The S_0_–T_1_ intersection depicted in Figure 4a describes a nonradiative deactivation pathway for the triplet state of complex **1**. The plot of the spin density on the iridium ion (Figure 4c) indicates that in the range of up to 40°, T_1_ is characterized by $d_{yz}/\pi_{oz}\to \pi_{oz}^{*}$ ; above 40°, its characteristics change to the d→d type. The characteristic of the triplet wave function can be discerned from the spin density contour: below 40°, the unpaired spin density is located mainly on the *oz* ligand and in part on iridium; above 50°, the unpaired electron density is found on the iridium ion. Figure 4d, shows that above the rotation angle of 40°, the Ir-N_*ox*_ bond undergoes rupture. In Figure S2, it can be seen that the Ir-N_*ox*_ bond is practically unchanged up to about 40°. As a result of further increase in the rotation angle, this distance grows rapidly, confirming the breakage of the Ir-N_*ox*_ bond.

#### 2.3.2. Rotation of the Phenolate Moiety

**Figure 5:**
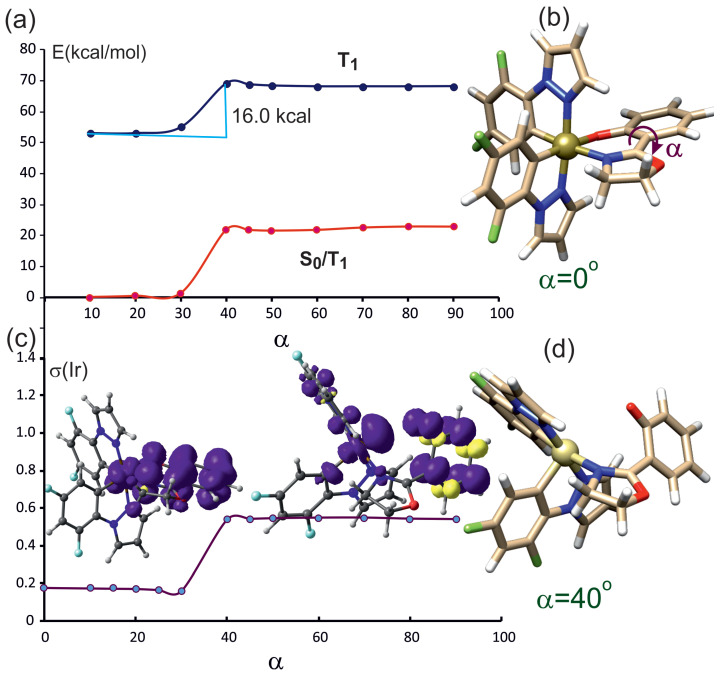
(**a**) Energy of the optimized triplet and singlet state in triplet geometry as a function of the rotation angle of the phenolate moiety. (**b**) Complex **1** with rotation angle 0°. (**c**) Spin density on the iridium ion as a function of the rotation angle. (**d**) Complex **1** with phenolate rotated 40°, where the Ir–O bond is broken and the transition between $d/\pi \to \pi^{*}$ to $d^{•}/phen^{•}$ takes place.

In Figure 5a, the PEC for the triplet state and singlet state in the triplet geometry as a function of the rotation angle of the phenolate group is presented. The spin density contours for the triplet state are shown in Figure 5c. They indicate that between rotation angles 30° and 40°, the triplet wave function changes its characteristic from $d/\pi_{oz}\to \pi_{oz}^{*}$ to the state where one electron resides on a *d* orbital of iridium and the second one resides on the oz ligand ( $d^{•}/phen^{•}$ ), as a consequence of phenolate oxygen–iridium homolytic bond breaking. The transition between these two triplet states involves an energy barrier of 16 kcal/mol. The energy of the singlet state is everywhere lower than that of the triplet state, and the curves do not intersect. The presented calculations show that the rotation of the phenolate group does not lead to the nonradiative deactivation of the triplet state in complex **1**.

### 2.4. Dimer of Ir(dfppz)_2_(oz)

**Figure 6:**
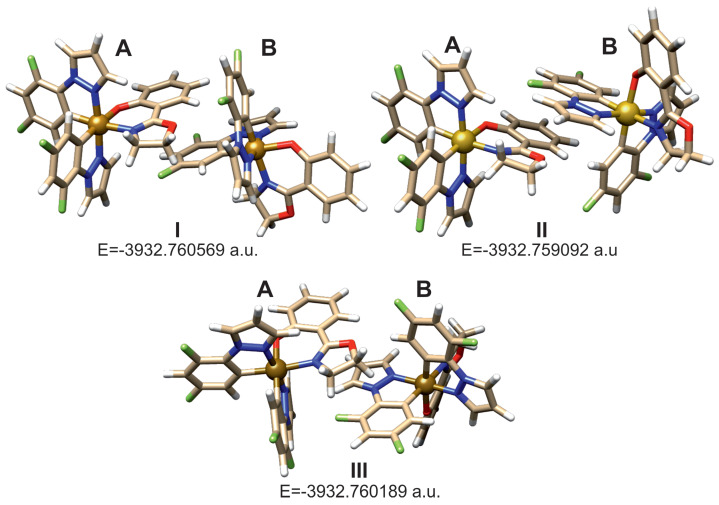
Optimized geometry of complex **1** dimeric structures.

The geometry of the three dimers of complex 1 were optimized, and their equilibrium structures are shown in Figure 6. A structural model was adopted in which the *oz* ligand from one molecule interacted through intermolecular forces with the *dfpp* ligand from the other, owing to which one *oz* ligand has the properties of a free molecule, and the one in which rotation takes place is under the influence of intermolecular forces. In this way, both *oz* ligands are in different situations, which allows the isolation of the influence of intermolecular forces on one *oz* ligand to not affect the other *oz* ligand.

Further calculations, the results of which are presented later in the text, were performed only for dimer **I**, which has the lowest energy; however, it can be noted that the energies for the optimized geometries of dimers are very close. The calculated interaction energy between the two molecules in the dimer was found to be 11.3 kcal/mol.

**Figure 7:**
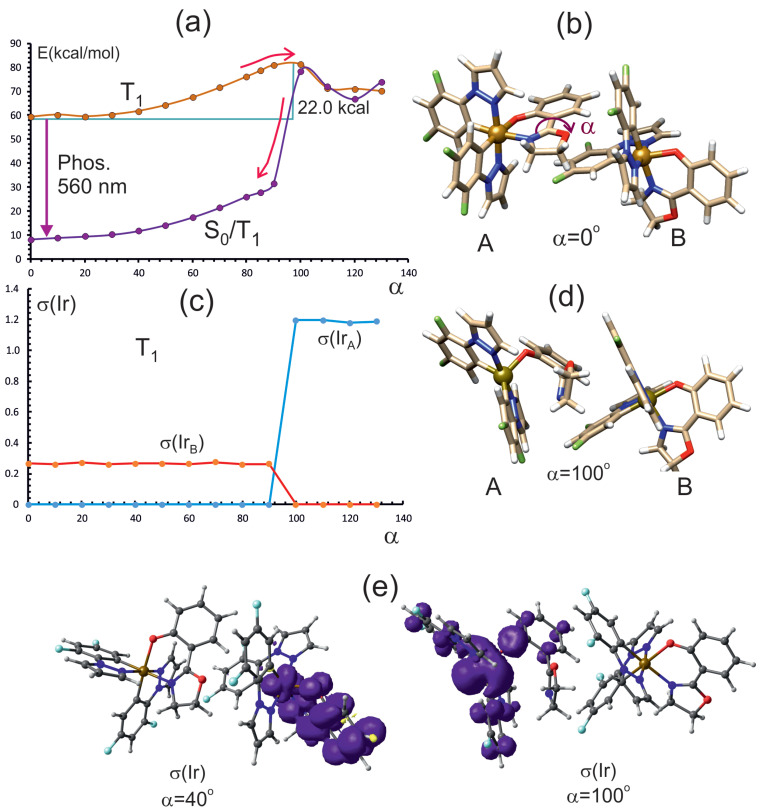
(**a**) Energy curves as function of oxozaline in unit **A** rotation angle: T_1_; the lowest energy singlet state calculated for the geometry of the triplet state (S_0_/T_1_. (**b**) Dimer geometry at the energy minimum. (**c**) Spin population on iridium ions as function of the rotation angle. (**d**) Dimer geometry at rotation angle of 100°, showing dissociated oxazoline moiety. (**e**) Spin density contours at rotation angles of 100° and 40°.

To determine the effect of intermolecular interactions on the energy of the dimer upon rotation within the *oz* ligand, which is in contact with the second molecule, we determined the energy of the triplet electronic state as a function of the rotation angle. The rotation of phenolate was not considered for the dimeric form, since it was shown as being inactive in deactivation process for the monomer. In Figure 7a, the potential energy curves for optimized geometries of the triplet state, T_1_, are presented as a function of the rotation of the oxazoline moiety in ligand *oz* of the **A** unit in dimer **I**. The energy of the singlet state calculated for the geometry of the triplet state (S_0_/T_1_) is also shown. The energy of the triplet rises with the rotation angle and reaches the maximum at an angle α of 100°. The maximum corresponds to the intersection of two triplet states. The respective energy barrier at the maximum equals 22.0 kcal/mol. The type of these two states can be deduced from the spin densities shown in Figure 7c,e. In the range of angles below 100°, thetriplet state is located on molecule **B** of the dimer and has a $d/\pi \to \pi^{*}$ character (Figure 7c,e). The spin density is located on the **B** molecule: partly on the iridium ion and mostly on the *oz* ligand. Its value on Ir_B_ is about 0.26. For the rotation angle starting from 100°, the triplet state is located on the **A** molecule of the dimer, mainly on the Ir ion. The spin density on Ir_*A*_ is about 1.2, which allows this electronic state to be classified as d→d type.

The singlet state energy calculated at the triplet geometry slowly rises up to a rotation angle of about 90°, then rises abruptly at about a 100° rotation angle, approaching the energy of the triplet function. Above 100°, the energies of both wave functions, singlet and triplet, are virtually the same. The singlet wave function approaches the triplet at that rotation angle, and the intersection between singlet and triplet requires the same energy as the maximum on the triplet energy curve, i.e., 22.0 kcal/mol. This barrier is much higher than in the monomer (8.4 kcal/mol), which is the cause of the radiationless deactivation path being blocked in the dimeric form. This situation is caused by the intermolecular interactions between two molecules in the dimer. As a consequence, the rotation of the oxazoline group is hindered. In Figure S2, the dependence of Ir-N_*ox*_ in the monomer and dimer is depicted. This distance in the dimer is virtually constant for rotation angles up to about 100°, where it increases very quickly. This corresponds to the breaking of the iridium–nitrogen bond and the transition to the d→d state. For the monomer, this process occurs much earlier, at about 40°.

## 3. Discussion

The AIEgen complex **1** studied in this work contains an *oz* ligand (2-(20-hydroxyphenyl)-2-oxazoline), which consists of two fragments—the oxazoline and phenolate moietes, linked by a C–C bond. To investigate the mechanism of AIE in this complex, we examined the rotation of the oxazoline and phenolate fragments around the bond connecting them. We also carried out a calculation for the dimeric form of the complex to clarify the effect of the intermolecular interactions on the luminescent properties of **1**.

### 3.1. Monomer Complex ***1***

Rotation of the oxazoline fragment in the triplet state leads to the intersection of the $d/\pi \to \pi^{*}$ state with the d→d state at a rotation angle (Figure 2) of about 40°. The bond with oxazoline is then broken, and the nitrogen atom moves away from the Ir cation. The iridium atom basically becomes five-coordinated; hence, it adopts a triplet configuration, effectively the d→d state. At the same time, the energy of the singlet ground state increases rapidly. The intersection of the singlet and the triplet provides an effective channel for the radiationless deactivation of the triplet to the ground state. On the other hand, the rotation of the phenolate part is associated with almost twice-higher barrier in the triplet zone. After breaking the Ir-O_*phen*_ bond, one electron is in the d orbital of the Ir ion, while the other is in the phenolate. The triplet and singlet states do not intersect—the singlet state is of much lower energy in this case. In general, it can be considered that nonradiative deactivation of the triplet state is rather impossible as a result of the rotation of the phenolate fragment.

**Figure 8:**
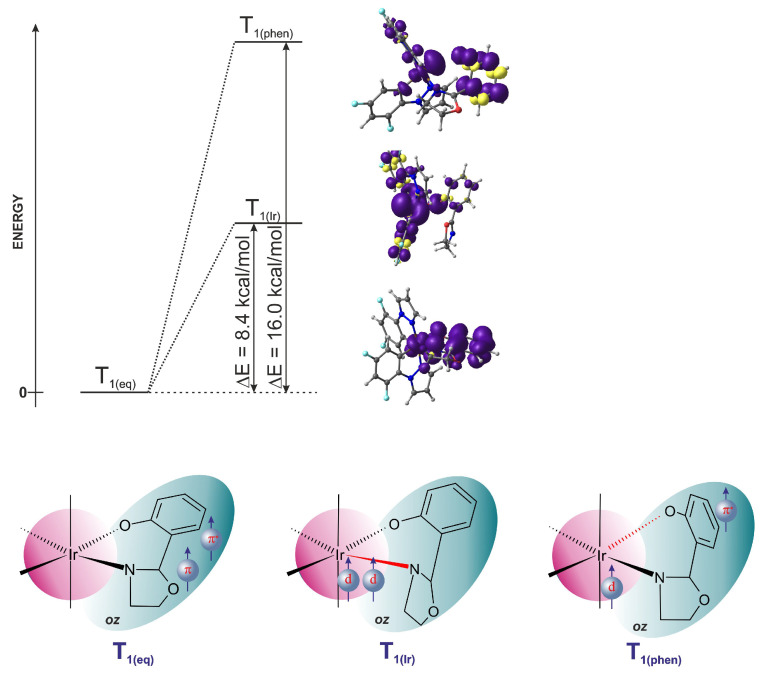
The three triplet state’s characteristics for complex **1**, from the left: equilibrium geometry, $d/\pi_{oz}\to \pi_{oz}^{*}$ state with two unpaired electrons mainly on the oxazoline fragment; d→d state at large rotation angle of oxazoline, with two electrons predominantly on iridium; triplet state at large rotation angle of the phenolate fragment, with one electron on iridium and the second one on phenolate.

The characteristics of the three triplet states relevant to the photophysical properties of **1** are depicted in Figure 8. It can be noticed that the spin populations on individual fragments of the molecule are smaller then one (Figure 4c and Figure 5c); hence, it is an idealized image of the spin density distribution in excited triplet states.

### 3.2. Dimer of Complex ***1***

To determine the effect of aggregation on the emission properties of complex **1**, we carried out calculations for the dimeric structure, where the *oz* ligand interacts with the *dfppz* ligand in two monomers. According to the results presented in Figure 7, the T_1_ energy has a maximum at an α of about 100°, which corresponds to the crossing of two wave functions of different character: $d/\pi_{ozB}\to \pi_{ozB}^{*}$ and $d_{A}\to d_{A}$ . This transition is caused by the following: at large rotation angles, the d→d state in the monomer **A** becomes lower in energy as the Ir-nitrogen bond breaks. At the same time, the energy of the singlet calculated for triplet geometry (S_0_/T_1_) increases and becomes practically equal with the triplet energy at the triplet maximum. The high barrier of this process of 22.0 kcal/mol effectively prevents the deactivation of the triplet state, leading to phosphorescence. The change in the triplet state nature is reflected in the spin densities on the iridium ions shown in Figure 7c,e, which is localized on the **B** monomer at rotation angles smaller than 100°, and on iridium in monomer **A** otherwise.

### 3.3. Mechanism of AIE in Complex ***1***

**Figure 9:**
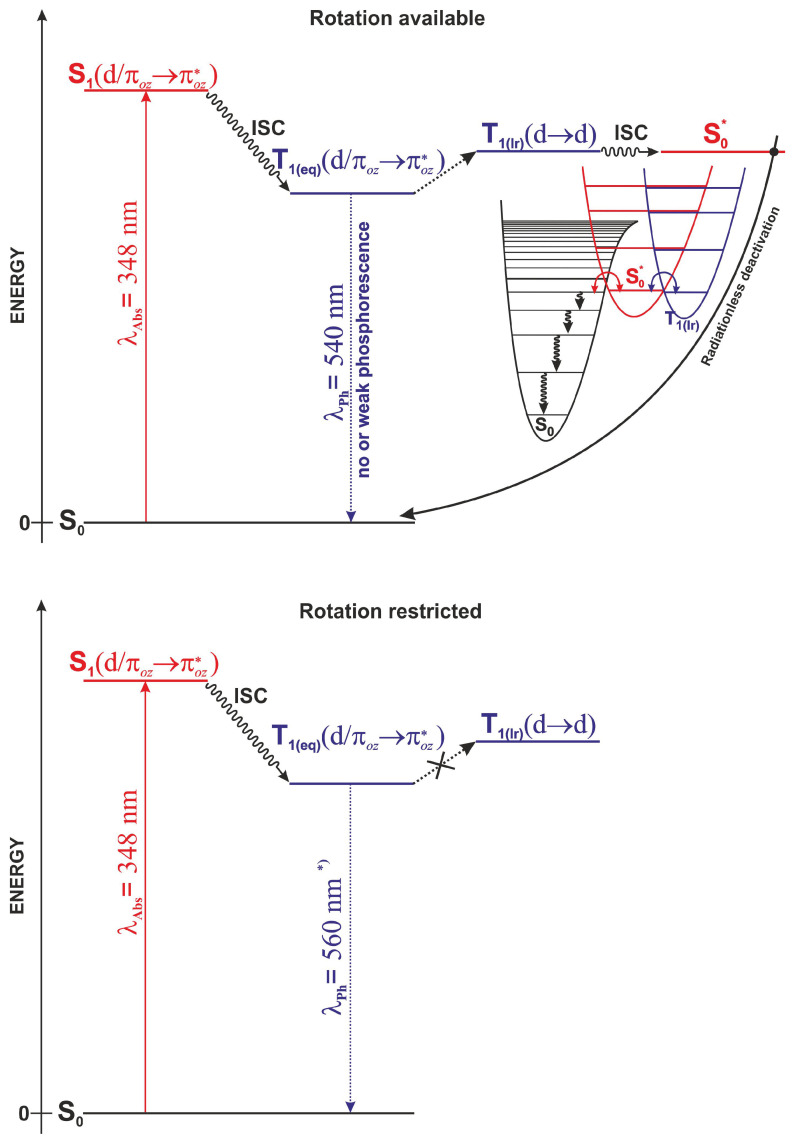
Mechanism of AIE in complex **1**.

Figure 9 shows a schematic diagram of the AIE mechanism in complex **1**, derived from the calculation results. After absorbing light of wavelength in the range of about 370 nm (calculated value 348 nm), the S_1_ state with a $d/\pi_{oz}\to \pi_{oz}^{*}$ characteristic can be populated directly or from a higher excited state via sequential IC processes. As a result of the ISC process, the S_1_ state undergoes conversion to a T_1_ triplet state with a similar $d/\pi_{oz}\to \pi_{oz}^{*}$ characteristic. After geometry relaxation, the T_1(eq)_ triplet is in the emissive state, from which the phosphorescence process can take place. However, the experiment data show that complex **1** in solution exhibits virtually no emission; therefore, a radiation-free deactivation path must be available. In the presented mechanism, the deactivation path is associated with the interaction between two triplet states $T_{1(\mathrm{eq})}\left(d/\pi_{oz}\to \pi_{oz}^{*}\right)⇝T_{1(\mathrm{Ir})}\left(d\to d\right)$ , as shown in the top panel in Figure 9. Such a process requires a deformation of the equilibrium geometry of the complex in the ligand region and/or within the coordination sphere. As previously noted, in the Ir complex, none of the ligands have substituents capable of free rotation; hence, we investigated the geometry deformation related to the intramolecular rotation of groups in the *oz* ligand around the C-C bond between the oxazoline (*ox*) and phenolate (*phen*) fragments. During rotation in the excited T_1(eq)_ triplet state of $d/\pi_{oz}\to \pi_{oz}^{*}$ type, at initial small values of the rotation angle, both fragments of the *oz* ligand most probably perform rotations up to 30–40° (Figure 4 and Figure 5). At larger rotation angles, it is energetically more favorable to twist the oxazoline fragment and, in effect, to detach nitrogen from the central atom, which leads to a change in the triplet state characteristic to the T_1(Ir)_ (d→d) type. The determined energy barrier for the transition between T_1(eq)_ and T_1(Ir)_ states is less than 8.5 kcal/mol. The T_1(Ir)_ triplet state is energetically near degenerate, with the $S_{0}^{*}$ singlet state in a twisted *oz* ligand geometry. Taking into account that the triplet and singlet are close in energy, the T_1(eq)_ ⇝ $S_{0}^{*}$ intersystem crossing should be effective, which means that the triplet state is quenched nonradiatively, and the system relaxes to the energy minimum of the S_0_ ground state and the equilibrium geometry of the complex (right-hand side of the panel in Figure 9). The second factor that causes the effectiveness of ISC is the fact that for large dihedral angles α , the potential energy curves of both states are almost parallel. This local surface topology suggests that around the PES intersection region, the difference in energy gradients of the interacting electronic states is small. This property, according to Landau–Zener theory [49], efficiently increases the probability of intersystem crossing.

To summarize, intramolecular rotation in the oxazoline ligand (oz) opens an additional triplet channel, competitive with phosphorescence, which leads to the nonradiative deactivation of the excited states. The photophysical mechanism of this channel is completely consistent with the experimental data, in which, in the absence of a possible rotation-blocking factor, a significant reduction in the intensity of phosphorescent radiation or its complete disappearance is observed. The most obvious factor blocking the rotation in the *oz* ligand is the intermolecular interactions that occur as a result of the formation of molecular aggregates. As can be seen from the calculations, already in the case of a dimer, the energy barrier for the transition between triplet states increases almost three times (calculated value of 22 kcal/mol, Figure 7). In such a situation, as shown in the bottom panel in Figure 9, the conversion between the T_1(eq)_ and T_1(Ir)_ states is blocked, and the emission-free triplet channel becomes inactive and noncompetitive with phosphorescence. Therefore, the mechanical blocking of rotation means that the deactivation of excited states can only occur as a result of photon emission. It is also worth noting that the results of the calculations indicate the possibility of luminescence batochromism. The calculated wavelength for phosphorescence for the monomer is 540 nm; for the dimer, it is 560 nm. The above-described mechanism of the AIE properties of **1** can be called restricted access to singlet–triplet crossing (RASTC).

## 4. Conclusions

For the complex under study, and low-energy deformation of the *oz* ligand structure and the Ir coordination sphere in the lowest triplet state cause the disappearance of phosphorescence, associated with the occurrence of a competitive, nonradiative triplet channel connecting the emission state with the electronic ground state. The partial or complete blocking of intramolecular movements due to aggregation of molecules blocks access to the nonradiative path and contributes to directing the photophysical process toward phosphorescence, giving rise to the AIE effect.

We have shown that the energy barrier that lead to the deactivation of the excited state and thus to the quenching of phosphorescence is approximately 2.5 times higher in the dimer than in the monomer of the iridium complex under study. The experimental magnitude of these barriers is unknown, so the calculated values cannot be verified. However, it seems that the model used explains the lack of luminescence in the monomer and its appearance in dimers and, further, in oligomers, where this phenomenon is enhanced.

## 5. Calculation Methods

The calculations were performed with the use of the Gaussian16 program [50]. The DFT method was used with the PBE0 functional [51].

The PBE0 functional is often used in calculations for transition metal complexes, including iridium complexes, and has been shown to accurately reproduce the electronic spectra and geometry of such compounds [52,53,54,55,56,57,58]. The def2-TZVP, (8s7p6d1f)/[6s4p3d1f], basis set with the appropriate ECP was used for iridium, while for carbon, nitrogen, and oxygen, the TZVP basis, (11s6p1d)/[5s3p1d] and (5s1p)/[3s1p], for hydrogen was used [59,60].

The SMD solvent model [61] was used with acetonitrile as the solvent. The GD3 dispersion corrections [62] were applied in the calculations. In order to determie the influence of the rotation within the *oz* ligand on the photophysical properties of complex **1**, the energy was determined as a function of the rotation angle C1-C2-C3-N_*ox*_ ( α , Figure 2), for the rotation of the oxazoline and phenolate fragments. The rotation angle was frozen with 10° increment, while other geometric parameters were optimized. The optimization for the dimeric forms of complex **1** was performed without any structural constraints. For the optimized structures of complex **1** and the dimer, all vibration frequencies were real.

The electronic spectra were calculated with the TD-DFT method. The energy of the lowest T_1_ state was calculated via independent optimization of the triplet spin wave function.

## Figures and Tables

**Table 1:** Selected geometry parameters of **1**. Bond lengths in Å, angles in degrees.

Parameter	Calc.	Expt. ^1^
Ir-O_phen_	2.122	2.131
Ir-N_*ox*_	2.112	2.122
Ir-N1	2.026	2.006
Ir-N2	2.016	1.987
Ir-C4	2.012	2.011
Ir-C5	1.998	1.996
C1-C2-C3-N_*ox*_	−6.9	−11.9

^1^ Ref. [44].

**Table 2:** TD-DFT lowest energy calculated electronic transitions of **1**. f and ω are the oscillator strength and weight of the given configuration, respectively.

E (eV)	λ (nm)	f	Characteristic	ω	Characteristic	ω	$\lambda_{\mathrm{ex}}$ (nm) ^1^
Absorption							
3.55	348.3	0.059	$H\left(d_{yz}/\pi_{oz}\right)\to L\left(\pi_{oz}^{*}\right)$	0.980			370
3.75	330.1	0.032	$H\left(d_{yz}/\pi_{oz}\right)\to L+1\left(\pi_{dfppz}^{*}\right)$	0.892			
3.80	326.2	0.015	$H\left(d_{yz}/\pi_{oz}\right)\to L+2\left(\pi_{dfppz}^{*}\right)$	0.919			
3.95	313.2	0.017	$H-1\left(d_{xy}/\pi_{dfppz}\right)\to L\left(\pi_{oz}^{*}\right)$	0.590	$H-1\left(d_{xy}/\pi_{dfppz}\right)\to L+1\left(\pi_{dfppz}^{*}\right)$	0.262	
4.00	309.9	0.026	$H-1\left(d_{xy}/\pi_{dfppz}\right)\to L+1\left(\pi_{dfppz}^{*}\right)$	0.583	$H-1\left(d_{xy}/\pi_{dfppz}\right)\to L\left(\pi_{oz}^{*}\right)$	0.336	325
4.06	304.9	0.010	$H-1\left(d_{xy}/\pi_{dfppz}\right)\to L+2\left(\pi_{dfppz}^{*}\right)$	0.828			
4.32	286.5	0.107	$H-2\left(d_{xz}\right)\to L\left(\pi_{oz}^{*}\right)$	0.621	$H-2\left(d_{xz}\right)\to L+1\left(\pi_{dfppz}^{*}\right)$	0.297	
4.37	283.2	0.052	$H-2\left(d_{xz}\right)\to L+1\left(\pi_{dfppz}^{*}\right)$	0.366	$H-2\left(d_{xz}\right)\to L+2\left(\pi_{dfppz}^{*}\right)$	0.328	290
			$H-2\left(d_{xz}\right)\to L\left(\pi_{oz}^{*}\right)$	0.264			
Phosphorescence							
2.30	540		$L\left(\pi_{oz}^{*}\right)\to H\left(d_{yz}/\pi_{oz}\right)$				500

^1^ Ref. [44].

## Data Availability

The results of the calculations are accessible from the repository at https://repod.icm.edu.pl/dataset.xhtml?persistentId=doi%3A10.18150%2FGN36VC, doi:10.18150/GN36VC.

## Supplementary Materials

The following supporting information can be downloaded at: https://www.mdpi.com/article/10.3390/molecules29030580/s1, Table S1. The twenty lowest vertical triplet electronic transitions for Ir(dfppz)_2_(oz) complex based on the TD-DFT/PBE0/def2-TZVP calculations with GD3 dispersion correction and SMD/acetonitrile solvent model, Table S2. The fifteen lowest vertical triplet electronic transitions for Ir(dfppz)_2_(oz) complex based on the TD-DFT/PBE0/def2-TZVP calculations with GD3 dispersion correction and SMD/acetonitrile solvent model, Figure S1. Molecular orbital diagram for complex **1**, Figure S2. Distance Ir-N_ox_ as a function of the oxazoline moiety rotation angle, Figure S3. Comparison of the conformation of complex **1** in the dimer and monomer for the geometry at the rotation angle at which the Ir-N_ox_ is broken, Figure S4. Superposition of optimized Ir(dfppz)_2_(oz) complex geometries for monomer and dimer. Presented geometries are form Figure S3.

## Abbreviations

The following abbreviations are used in this manuscript:

AIE	Aggregation Induced Emission
PEC	Potentia Energy Curve
RASTC	Restricted Acess to Singlet-Triplet Crossing
ISC	Intersystem Crossing
IC	Internal Conversion
