# Supplementary material for: Theoretical Investigation of Iridium Complex with Aggregation-Induced Emission Properties
Source: Molecules. 2024 Jan 24;29(3):580. doi: 10.3390/molecules29030580 (PMC10856369; doi:10.3390/molecules29030580)
Supplement: Supplementary file 1 [file molecules-29-00580-s001.zip › molecules-2783973-supplementary.pdf]

Table S1. The twenty lowest, vertical triplet electronic transitions for Ir(dfppz)$_2$(oz) complex based on the TD-DFT/PBE0/def2-TZVP calculations with GD3 dispersion correction and SMD/acetonitrile solvent model.

E(eV)	λ(nm)	f	Character	ω	Character	ω	λ_{ex}(nm)[1]
3.55	348.3	0.059	$H(d_{yz}/\pi_{oz}) \rightarrow L(\pi^*_{oz})$	0.980			370
3.75	330.1	0.032	$H(d_{yz}/\pi_{oz}) \rightarrow L+1(\pi^*_{dfppz})$	0.892			
3.80	326.2	0.015	$H(d_{yz}/\pi_{oz}) \rightarrow L+2(\pi^*_{dfppz})$	0.919			
3.95	313.2	0.017	$H-1(d_{xy}/\pi_{dfppz}) \rightarrow L(\pi^*_{oz})$	0.590	$H-1(d_{xy}/\pi_{dfppz}) \rightarrow L+1(\pi^*_{dfppz})$	0.262	
4.00	309.9	0.026	$H-1(d_{xy}/\pi_{dfppz}) \rightarrow L+1(\pi^*_{dfppz})$	0.583	$H-1(d_{xy}/\pi_{dfppz}) \rightarrow L(\pi^*_{oz})$	0.336	325
4.06	304.9	0.010	$H-1(d_{xy}/\pi_{dfppz}) \rightarrow L+2(\pi^*_{dfppz})$	0.828			
4.32	286.5	0.107	$H-2(d_{xz}) \rightarrow L(\pi^*_{oz})$	0.621	$H-2(d_{xz}) \rightarrow L+1(\pi^*_{dfppz})$	0.297	
4.37	283.2	0.052	$H-2(d_{xz}) \rightarrow L+1(\pi^*_{dfppz})$	0.366	$H-2(d_{xz}) \rightarrow L+2(\pi^*_{dfppz})$	0.328	290
			$H-2(d_{xz}) \rightarrow L(\pi^*_{oz})$	0.264			
4.45	278.0	0.069	$H-2(d_{xz}) \rightarrow L+2(\pi^*_{dfppz})$	0.521	$H-2(d_{xz}) \rightarrow L+1(\pi^*_{dfppz})$	0.245	
4.49	275.9	0.064	$H(d_{yz}/\pi_{oz}) \rightarrow L+3(\pi^*_{dfppz})$	0.825			
4.61	268.5	0.013	$H-4(d_{yz}/\pi) \rightarrow L(\pi^*_{oz})$	0.683			
4.63	267.2	0.008	$H(d_{yz}/\pi_{oz}) \rightarrow L+4(\pi^*_{dfppz})$	0.648			
4.70	263.4	0.013	$H-1(d_{xy}/\pi_{dfppz}) \rightarrow L+3(\pi^*_{dfppz})$	0.511			
4.78	259.1	0.075	$H-4(d_{yz}/\pi) \rightarrow L+1(\pi^*_{dfppz})$	0.227	$H-3(\pi_{dfppz}) \rightarrow L+1(\pi^*_{dfppz})$	0.161	
			$H-1(d_{xy}/\pi_{dfppz}) \rightarrow L+3(\pi^*_{dfppz})$	0.220			
4.83	256.2	0.005	$H-3(\pi_{dfppz}) \rightarrow L(\pi^*_{oz})$	0.865			
4.86	254.6	0.021	$H-3(\pi_{dfppz}) \rightarrow L+1(\pi^*_{dfppz})$	0.378	$H-4(d_{yz}/\pi) \rightarrow L+1(\pi^*_{dfppz})$	0.306	
4.87	254.1	0.082	$H-4(d_{yz}/\pi) \rightarrow L+2(\pi^*_{dfppz})$	0.351	$H-3(\pi_{dfppz}) \rightarrow L+1(\pi^*_{dfppz})$	0.345	
4.91	252.2	0.199	$H-3(\pi_{dfppz}) \rightarrow L+2(\pi^*_{dfppz})$	0.622	$H-4(d_{yz}/\pi) \rightarrow L+2(\pi^*_{dfppz})$	0.131	260
4.96	249.9	0.082	$H-1(d_{xy}/\pi_{dfppz}) \rightarrow L+4(\pi^*_{dfppz})$	0.291	$H(d_{yz}/\pi_{oz}) \rightarrow L+5(\pi^*_{oz})$	0.172	

Table S2. The fifteen lowest, vertical triplet electronic transitions for Ir(dfppz)$_2$(oz) complex based on the TD-DFT/PBE0/def2-TZVP calculations with GD3 dispersion correction and SMD/acetonitrile solvent model.

	E(eV)	λ(nm)	ω(%)	MOs	Character	
T_1	2.81	441	90	142 → 143	H → L	d/π_{oz} → π_{oz}*
T_2	3.27	379	14	142 → 144	H → L+1	d/π_{oz} → π_{dfppz}*
			14	141 → 144	H-1 → L+1	d/π_{dfppz} → π_{dfppz}*
			12	139 → 144	H-3 → L+1	π_{dfppz} → π_{dfppz}*
			12	139 → 145	H-3 → L+2	π_{dfppz} → π_{dfppz}*
T_3	3.29	377	18	141 → 145	H-1 → L+2	d/π_{dfppz} → π_{dfppz}*
			12	139 → 144	H-3 → L+1	π_{dfppz} → π_{dfppz}*
			12	141 → 144	H-1 → L+1	d/π_{dfppz} → π_{dfppz}*
T_4	3.34	371	26	140 → 143	H-2 → L	d/π_{oz}/π_{dfppz} → π_{oz}*
			22	135 → 143	H-7 → L	π_{oz}/π_{dfppz} → π_{oz}*
T_5	3.53	352	28	142 → 144	H → L+1	d/π_{oz} → π_{dfppz}*
			14	142 → 145	H → L+2	d/π_{oz} → π_{dfppz}*
T_6	3.62	342	22	142 → 145	H → L+2	d/π_{oz} → π_{dfppz}*
			22	140 → 144	H-2 → L+1	d/π_{oz}/π_{dfppz} → π_{dfppz}*
			20	140 → 145	H-2 → L+2	d/π_{oz}/π_{dfppz} → π_{dfppz}*
T_7	3.79	327	26	142 → 148	H → L+5	d/π_{oz} → π_{oz}*
			22	141 → 143	H-1 → L	d/π_{dfppz} → π_{oz}*
T_8	3.84	323	20	142 → 144	H → L+1	d/π_{oz} → π_{dfppz}*
			20	142 → 145	H → L+2	d/π_{oz} → π_{dfppz}*
T_9	3.90	318	20	141 → 144	H-1 → L+1	d/π_{dfppz} → π_{dfppz}*
			10	141 → 145	H-1 → L+2	d/π_{dfppz} → π_{dfppz}*
T_{10}	3.95	314	42	141 → 143	H-1 → L	d/π_{dfppz} → π_{oz}*
			18	140 → 143	H-2 → L	d/π_{oz}/π_{dfppz} → π_{oz}*
T_{11}	3.98	311	30	141 → 145	H-1 → L+2	d/π_{dfppz} → π_{dfppz}*
			12	141 → 144	H-1 → L+1	d/π_{dfppz} → π_{dfppz}*
T_{12}	4.06	305	12	141 → 146	H-1 → L+3	d/π_{dfppz} → π_{dfppz}*
			10	141 → 145	H-1 → L+2	d/π_{dfppz} → π_{dfppz}*
			8	142 → 146	H → L+3	d/π_{oz} → π_{dfppz}*
T_{13}	4.08	304	18	141 → 147	H-1 → L+4	d/π_{dfppz} → π_{dfppz}*
			12	137 → 145	H-5 → L+2	π_{dfppz}/d → π_{dfppz}*
			12	141 → 146	H-1 → L+3	d/π_{dfppz} → π_{dfppz}*
			10	137 → 144	H-5 → L+1	π_{dfppz}/d → π_{dfppz}*
T_{14}	4.14	300	12	141 → 147	H-1 → L+4	d/π_{dfppz} → π_{dfppz}*
			8	138 → 144	H-4 → L+1	d/π_{oz}/π_{dfppz} → π_{dfppz}*
			8	140 → 144	H-2 → L+1	d/π_{oz}/π_{dfppz} → π_{dfppz}*
			8	140 → 145	H-2 → L+2	d/π_{oz}/π_{dfppz} → π_{dfppz}*
			8	141 → 146	H-1 → L+3	d/π_{dfppz} → π_{dfppz}*
T_{15}	4.30	288	28	135 → 143	H-7 → L	π_{oz}/π_{dfppz} → π_{oz}*
			16	142 → 148	H → L+5	d/π_{oz} → π_{oz}*
			14	133 → 143	H-9 → L	π_{oz}/π_{dfppz} → π_{oz}*

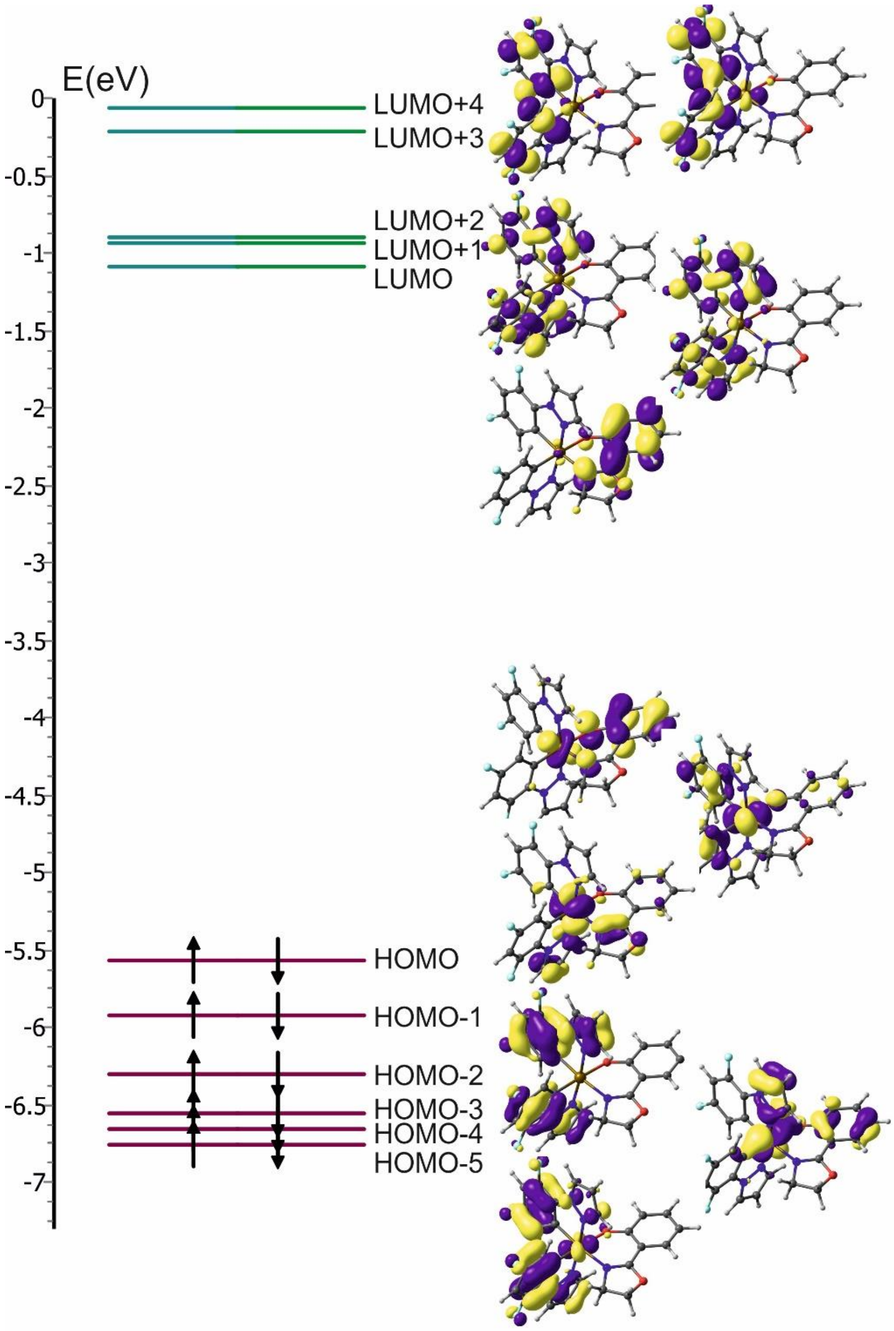

Figure S1. Molecular orbital diagram for complex **1.**

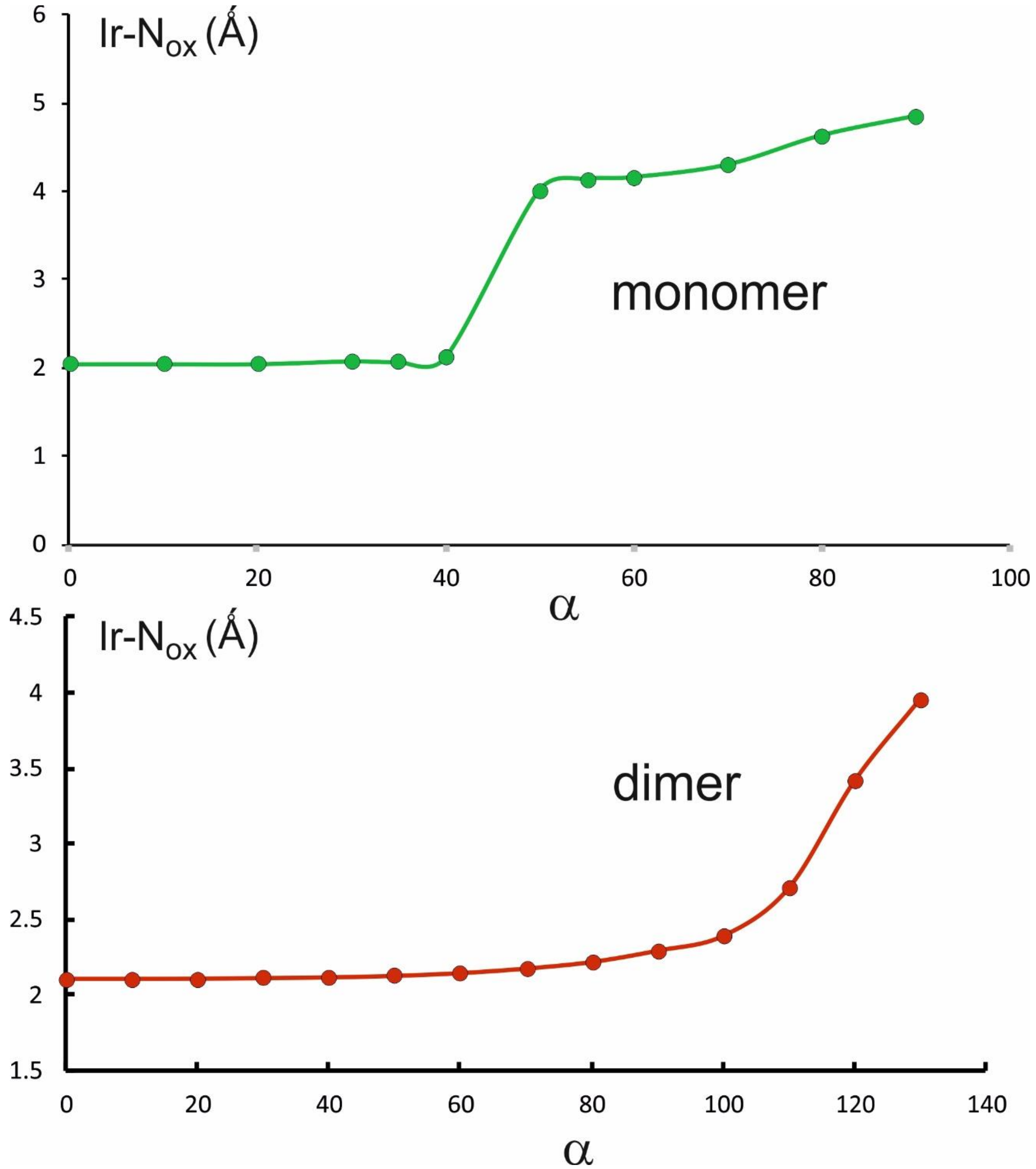

Figure S2. Distance Ir-N$_{ox}$ as a function of the oxazoline moiety rotation angle.

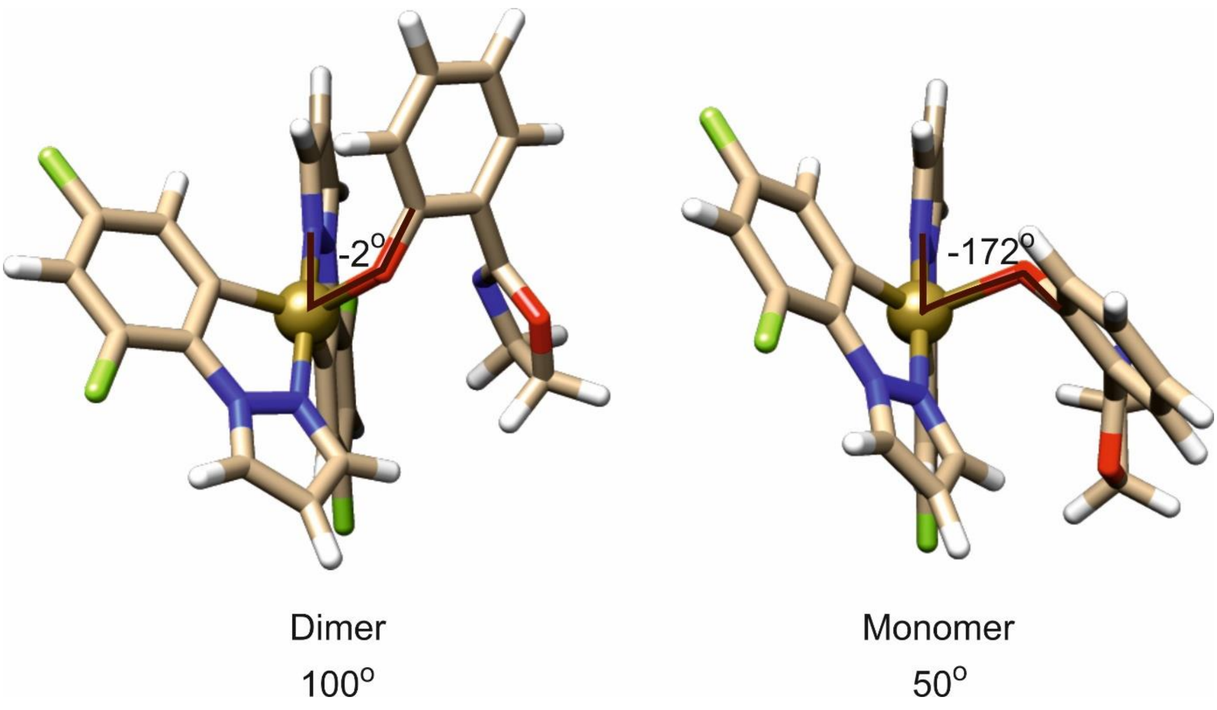

Figure S3. Comparison of the conformation of complex 1 in the dimer and monomer for the geometry at the rotation angle at which the Ir-N_{ox} is broken.

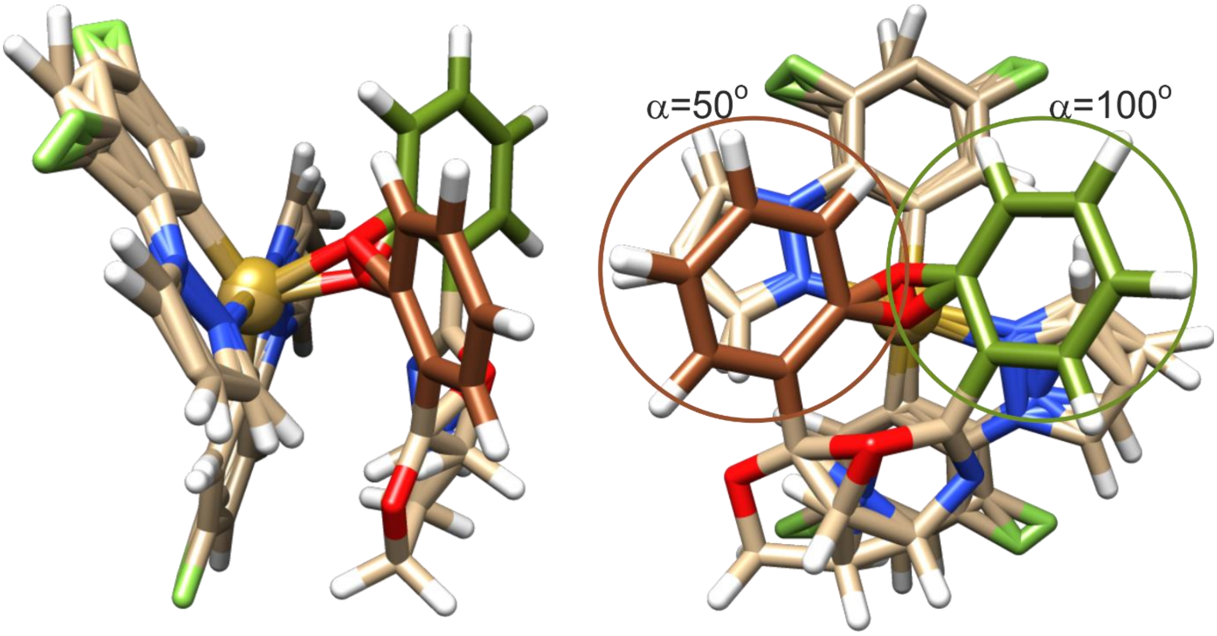

Figure S4. Superposition of optimized Ir(dfppz)$_2$(oz) complex geometries for monomer and dimer. Presented geometries are form Figure S3.
